# Morningness and Conscientiousness: A Meta-analysis, Online Survey and Resting fMRI Study

**DOI:** 10.5334/jcr.240

**Published:** 2025-04-07

**Authors:** Debore Buzoku, Sahar Esmat, Ray Norbury

**Affiliations:** 1 Department of Life Sciences, Division of Psychology, Centre for Cognitive and Clinical Neuroscience, College of Health, Medicine and Life Sciences, Brunel University London, United Kingdom

**Keywords:** Chronotype, Conscientiousness, Meta-analyses, Resting fMRI

## Abstract

Morningness is associated with several positive health outcomes and personality traits such as conscientiousness. In the current report, meta-analysis demonstrated a significant association between morningness and conscientiousness, data that are consistent with previous meta-analyses. Further, survey report and resting-state functional Magnetic Resonance Imaging (rs-fMRI, *N* = 43) indicated that the relationship between morningness and conscientiousness was moderated by functional connectivity with the Default Mode Network (DMN). DMN connectivity has been implicated in a number of cognitive functions and higher connectivity in this network is associated with higher conscientiousness.

## Introduction

Diurnal preference (an individual’s preference for timing of daily activities) varies along a continuum ranging from morning-type individuals (“larks”) who rise early, are more active and alert in the earlier part of the day and retire to bed early; and evening-types, who prefer to rise later in the day, are more active and alert in the later part of the day and tend to retire to bed late.

Objective measures of circadian rhythms include actigraphy and core body temperature, but reliable self-report scales of diurnal preference are of greater utility for research purposes, especially with large samples. Among the most frequently used measures are the Morningness-Eveningness Questionnaire (MEQ) [1] and it’s shorter counterpart the Reduced MEQ (rMEQ) [2]. The full version of the MEQ includes 19 items related to habitual rise and bed times, preferred times for physical and mental activity and subjective alertness immediately after rising and before going to bed. Other widely used measures include the 13-item Composite Scale of Morningness (CSM) [3], the Morningness-Eveningness-Stability-Scale improved (MESSi) [4] and the Caen Chronotype Questionnaire (CCQ) [5].

Diurnal preference has been linked to a number of physical and mental health outcomes. For example, a more evening profile has been associated with type-2 diabetes and cardiovascular disease [6], psychiatric disorder [7,8,9] and depressive symptomatology [10]. Related to physical and mental health, evidence also suggests that evening-type individuals are more likely to engage with stimulants such as caffeine, alcohol or cigarettes [11,12] and show greater use of smart devices and internet addiction [13].

In addition to physical and mental health, a number of studies have also examined the relationship between diurnal preference and personality traits, initially with a focus on Eysenck’s three-factor model (i.e. PEN-model: extraversion, neuroticism and psychoticism) [14] but more recently focusing on the five-factor model (FFM) of personality as a theoretical framework [15]. The FFM (also referred to as the Big-Five) comprises: 1) Openness to experience, broadly defined as the tendency to be open to new feelings, thoughts and values, 2) Conscientiousness, the tendency to be systematic, goal-orientated and industrious, 3) Extraversion, the tendency to be out-going, social and energetic, and 4) Agreeableness, the tendency to cooperative, sympathetic and trusting [16,17,18]. Using the FFM framework, two meta-analyses [16,18], together spanning 70 samples including over 26,000 participants, reported mild to moderate relationships between diurnal preference and the big-five personality traits, with the strongest relationship between diurnal preference and conscientiousness. Following from these data, the first aims of the current study were: 1) To extend previous meta-analyses to include more recent research (January 2018–December 2022), and 2) Conduct an online survey in healthy participants to demonstrate the relationship between diurnal preference and conscientiousness.

The neural substrates of conscientiousness have been examined using techniques including structural Magnetic Resonance Imaging (sMRI) and functional MRI (fMRI). Using sMRI, a number of studies have reported positive correlations between conscientiousness and grey matter volume in lateral prefrontal cortex [19,20,21,22] right orbitofrontal cortex and right putamen [20,22], and negatively correlated with insula cortex [20,23,24]. Using fMRI Dima and colleagues [25] demonstrated that effective connectivity between parietal and dorsolateral prefrontal cortex was positively related to conscientiousness.

Besides exploring personality measures in terms of anatomy or activation in isolated brain regions, it is also possible to investigate brain networks (whole-brain circuitry) using resting-state MRI (rsMRI). Taking this approach, and using a large open-source data set (the Human Connectome Project) [26]. Toschi *et al*., [27] reported greater functional connectivity in DMN in participants higher in conscientiousness, which the authors suggested contributes to the observation that individuals high in conscientiousness perform well in terms of goal-setting and planning [27]. More recently, Cai and colleagues [28] using a machine-learning approach (connectome-based predictive modelling [CPM]) reported that connectivity within a negative network (i.e. edge parameters that negatively correlated with behavioural measures – DMN) significantly predicted conscientiousness. Current evidence suggests that individual differences in connectivity in DMN contribute to variability in measures of conscientiousness [27,28]. A further aim of this study, therefore, was to determine if connectivity within the DMN moderated the link between diurnal preference and conscientiousness. It was hypothesised that the relationship between diurnal preference and consciousness will be greater in individuals with higher functional connectivity within the DMN.

## Methods

### Meta-analysis

#### Literature review

PubMed and Web of Science were searched for articles published between 1^st^ January 2018 and 31^st^ December 2022 using the search terms “chronotype” OR “diurnal preference” OR “circadian preference” OR “morningness” OR “eveningness” OR “social jetlag” OR “chronopsychology”) AND (“personality” OR “neuroticism” OR “extraversion” OR “five factor” OR “neo” OR “conscientious” OR “openness” OR “aggreeableness” OR “dark triad”). The titles and abstracts of articles returned using this search were initially screened before the full text was examined in greater detail.

Inclusion criteria for the meta-analysis were: 1) Articles published in peer-reviewed journals (note, we also included data from the current report), 2) Sufficient information to estimate an effect size, 3) Written in English, 4) Text freely available, 5) Includes only adult participants, 6) Diurnal preference and conscientiousness estimated using a standardised instrument (details of the measures included are provided in Table 1).

**Table 1:** Study details (including the current online data) included in the initial random effects analysis. Chronotype measure: rMEQ = Morningness Eveningness a Reduced Scale, MESSi = Morningness-Eveningness-Stability-Scale improved (sign reversed as higher scores indicate increasing eveningness), MEQ = Morningnesss Eveningness Questionnaire, CSM = Composite Scale of Morningness CCQ = Caen chronotype questionnaire (sign reversed as higher scores indicate increasing eveningness). Personality measure: BFI = Big Five Inventory, BFI –44 = Big Five Inventory (44 Item), BFI-10 = Big Five Inventory (10 Item), BFI-Fr = BFI = Big Five Inventory French translation, IGFP-5 = Inventory of the five great personality factors, TIPI = The Ten-Item Personality Inventory, IPIP-BFM-50 = International Personality Item Pool big Five Markers 50-item, IPIP-BFM-20 = International Personality Item Poolbig Five Markers 20-item, IPIP-NEO-PI-R = International Personality Item Pool of the Revised NEO Personality Inventory, IPIP-50-Big-Five = International Personality Item Pool Five-Factor questionnaire, NEO-FFI = NEO-Five Factor Inventory.


AUTHOR	N	CHRONOTYPE MEASURE	PERSONALITY MEASURE	SAMPLE	MEAN AGE	AGE RANGE	FEMALE (%)

Buzoku, 2023	43	rMEQ	BFI	Students	24	18–48	67

Carcifo, 2022b	369	MESSi	BFI-44	Students	19	18–30	71

Santos, 2022	79	MEQ	IGFP-5	Students	21	18–44	73

Stevenson, 2022	864	MEQ	TIPI	General population	23	18–76	78

Gorgol, Stolarski, et al., 2022	380	CSM	IPIP-NEO-PI-R	General population	27	18–35	51

Gorgol,Waleriaczyk, et al., 2022	913	CSM	IPIP-NEO-PI-R	General population	26	18–35	51

Carcifo, 2022a	265	MESSi	BFI-10	Students	21	18–33	64

Przepiorka, 2021	398	CSM	IPIP-BFM-20	Students	20	18–30	71

Schredl, 2020	2492	MEQ	NEO-FFI-30	General population	48	18–93	58

Milic, 2020	712	rMEQ	IPIP-50-Big-Five	Students	23	NR	72

You, 2020	362	CCQ	BFI-Fr	Students	21	18–29	85

Carcifo, 2019	767	rMEQ	BFI-10	Students	20	18–50	71

Zajenkowski et al, 2019	505	CSM	IPIP-BFM-50	General population	38	18–70	61

Drezno et al, 2018	379	CSM	IPIP-BFM-50	General population	36	18–69	66

Faßl et al, 2018	97	MESSi	NEO-FFI	Students	25	18–54	75


#### Data analysis

Statistical analyses were performed using R version 3.6.1 [29] including the following packages: esc; effectsize; meta; metafor; dmetar: DiagrammeR; DiagrammeRsvg; and ggplot2. Individual effect sizes were obtained from each study (correlation coefficients), and the Fisher’s Z transformed correlation coefficients entered as the summary effect size. The corresponding pooled effect size and its 95% confidence intervals (CI) were calculated from a random-effects model with a Sidik–Jonkman estimator for τ^2^ with Hartung–Knapp adjustment. This method was adopted as a conservative approach in the presence of sample heterogeneity [30]. A pooled effect size of 0.1–0.3 was considered small, 0.3–0.5 medium and 0.5–1 considered a large effect. Study heterogeneity was assessed with Q statistics and the *I*^2^ index. Outlier analysis (studies were considered outliers if the 95% CI was outside the pooled effect size 95% CI) were also performed and the random-effects model refitted after excluding any such study. Categorical moderators (student sample *vs*. general population) were investigated using subgroup analyses. Continuous variables (age and percentage female participants) were examined using meta-regression.

### Survey

#### Participants

A total of 43 participants (Mean age = 23.21, SD = 6.36, range 18–47, 29 females) completed an online survey presented using Qualtrics (Qualtrics^®^, Provo, UT). The survey was available for completion between 01/08/2021 and 01/06/2022 and participants were free to complete the survey at a time convenient for them (but within 48 hours of fMRI scan – see below for scanning details). Exclusion criteria were age <18 and current or previous diagnosis of any psychiatric disorder.

#### Materials

Conscientiousness was determined using the 9 items from the Big Five Inventory, 44-item scale [31]. Each item was scored on a 1–5 (1 = disagree strongly, 5 = agree strongly). Likert-type scale with higher scores indicating more conscientiousness. Diurnal preference was measured using the reduced version of the Morningness-Eveningness questionnaire [2]. This is a five-item instrument with higher scores indicating greater morningness.

#### Data analysis

Visual inspection of Q-Q plots and Shapiro-Wilk tests were used to test assumptions of approximately normally distributed data. Simple Pearson’s bivariate correlation was used to determine the relationship between diurnal preference and conscientiousness.

### Resting fMRI

#### Participants

The same participants that completed the online survey (please see above for details) also completed the resting fMRI scan. Exclusion criteria as above and including any contra-indication for MRI examination.

#### Image acquisition

Imaging data were acquired on a research dedicated 3T Magnetom Trio (Siemens, Erlangen, Germany) fitted with a 32-channel head coil and located at the Combined Universities Brain Imaging Centre (CUBIC). For each participant we collected a T_1_-weighted whole brain scan (magnetization-prepared rapid acquisition with gradient echo (MPRAGE), inversion time (TI) = 1100 ms, repetition time (TR) = 1830 ms, echo time (TE) = 3.03 ms, flip angle (FA) = 11°, field of view (FOV) = 256 × 256 × 160 mm^3^, voxel size = 1 × 1 × 1 mm^3^). Resting MRI (rsMRI) data were acquired using a T_2_^*^-weighted echo planar imaging sequence (EPI, TR = 3000 ms, TE = 31 ms, FA = 85°, FOV = 192 × 192 × 126 mm^3^ [42 slices, voxel size = 3 × 3 × 3 mm^3^], number of measurements = 200, imaging bandwidth = 752 Hz/px, GRAPPA acceleration factor = 2). Gradient echo field mapping data were also acquired for EPI off-resonance distortion correction (TR = 400 ms, TE1 = 5.19 ms, TE2 = 7.65 ms, flip angle = 60°, FOV = 192 × 192 × 126 mm^3^, voxel size = 3 × 3 × 3 mm^3^). The resting-state scan was 10 minutes in duration and participants were instructed to remain awake and let their mind wander. For the duration of the scan a central fixation cross was presented to participants and viewed via an angled mirror mounted on the head coil.

#### Image preprocesing

All image pre-processing and analyses were performed using FSL version 6.0.10 (FMRIB Software Library, http://fsl.fmrib.ox.ac.uk/fsl/fslwiki/). The following pre-statistical processes were applied: 1) Anatomical data – T_1_-weighted images were bias field corrected and stripped to remove non-brain material, 2) rsMRI data: non-brain removal; rigid-body motion correction; high-pass temporal filtering (Gaussian-weighted least-squares fitting with frequency cut-off point = 100 s) and correction of off-resonance geometric distortions in the EPI data using B0 field maps derived from the dual echo gradient echo dataset. FIX (FMRIB’s ICA-based X-noisefier) was used to remove the artefactual components that reflected non-neuronal fluctuations. FIX was trained using a trained weights file generated from hand-labelling 46 participants from a previous study (Horne & Norbury, 2018) acquired on the same scanner using the same MR sequence. The pre-processed and cleaned rsMRI scans were non-linearly registered to standard space and spatially smoothed using a Gaussian kernel of 5 mm full width at half at half maximum (FWHM).

#### Group Independent Components Analysis (ICA) and statistical inference

**Figure 1:**
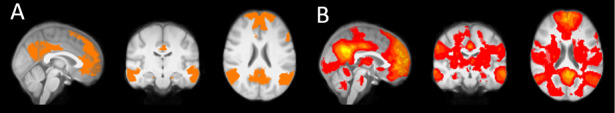
Left image shows the DMN determined by Yeo (2011), on the right the DMN determined from the current data (N = 43). Image is in neurological format (left brain on the right side), sagittal, coronal and axial views displayed.

Group ICA was performed using Multivariate Exploratory Linear Optimized Decomposition into Independent Components (MELODIC) with 25 dimensions (low dimensionality, <30, is optimal to estimate large scale resting networks). To determine the study-specific DMN each component from the previous step was spatially cross-correlated with the DMN reference map reported by Yeo and colleagues [33] and the component with the highest spatial overlap (*r* = 0.53, please see Figure 1.) identified as the study-specific DMN. Dual regression was then used to derive subject-specific maps for subsequent statistical analysis. Briefly, the study-specific DMN template was used in a general linear model fit (as spatial regressors) against the rsMRI preprocessed data, the output being the corresponding temporal dynamics for each subject. Secondly, these time courses were used in a second general linear model (as temporal regressors) against the rsMRI data to estimate a subject-specific spatial map. As a result, the values in these maps (parameter estimates, PE) represent the connectivity of each voxel with the DMN. At the group level, DMN connectivity was tested for significance using non-parametric permutation tests (applying 5000 permutations) and control for the Type-1 error rate obtained using threshold-free cluster enhancement (TFCE). The potential moderating effect of DMN connectivity on the relationship between diurnal preference and conscientiousness was explored using regression analysis. Finally, we used the Johnson-Neyman technique to identify the regions of significance for the observed interaction.

#### Ethical approval

Prior to any study procedures taking place (online survey and neuroimaging) full ethical approval was obtained from the College of Health, Medicine and Life Sciences Research Ethics Committee, Brunel University London (Reference: 27325-MHR-Jun/2021-33168–4).

## Results

### Meta-analysis

**Figure 2:**
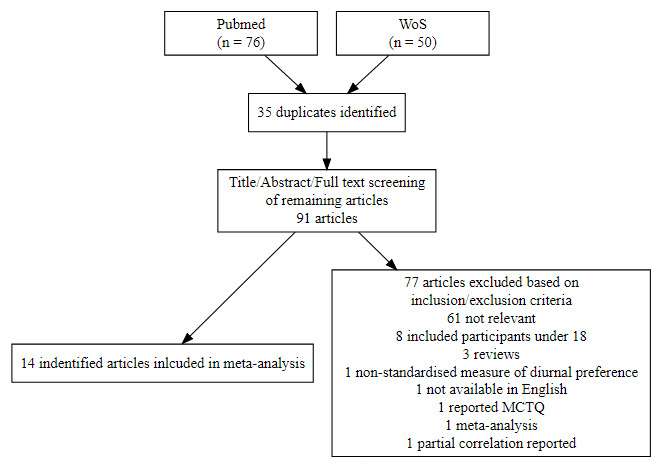
Study selection process. WoS = Web of science. Numbers in parentheses show original articles identified.

The initial literature search completed using the search terms listed above returned a total of 126 articles (PubMed = 76). Following article screening (see Figure 2 for a graphical overview, Table 1 for included study details) a total of 15 studies (including the current data) were entered into the initial random effects model [34,35,36,37,38,39,40,41,42,43,44,45,46,47].

**Figure 3:**
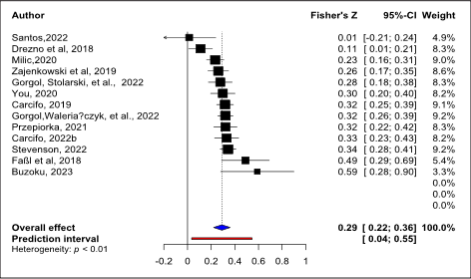
Forest plot of standardised effect sizes from each study. The overall effect is indicated in blue, prediction interval in red.

Outlier analyses identified two studies [34,44] and both were excluded from the main analysis. Heterogeneity was reduced after excluding these studies (prior to exclusion *I*^2^ = 94.2%, Q(14) = 241.77, *p* < 0.001, after exclusion *I*^2^ = 63.5%, Q(12) = 32.85, *p* .001) but remained substantial and significant. The final sample included a total of 13 effect sizes obtained from 5868 participants with a mean age of 25 years. The effect size estimated from the random-effects model was Fisher’s *Z* = 0.29, 95% CI [0.22–0.36], *p* < 0.001, demonstrating a small to moderate association between diurnal preference and conscientiousness (please see Figure 3, Forest plot summarising the above data).

**Figure 4:**
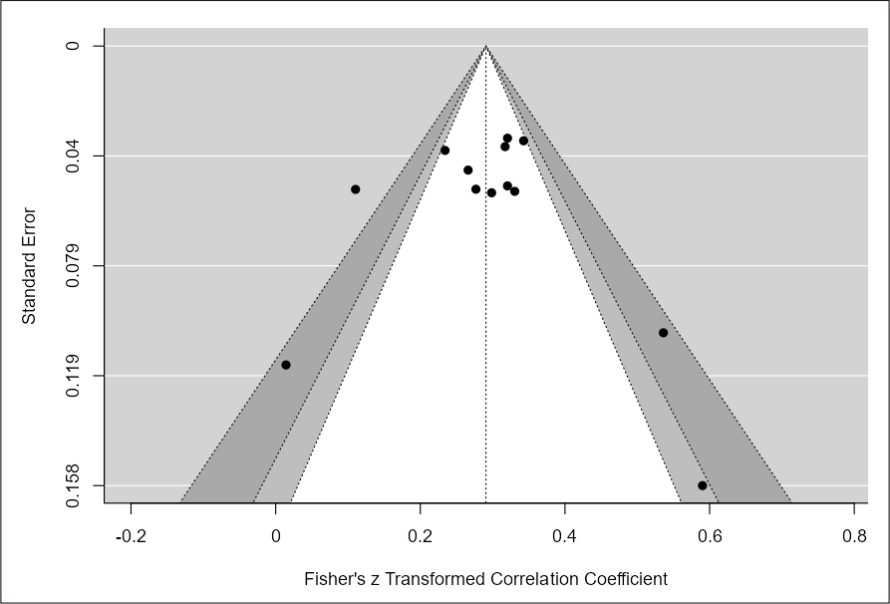
Contour funnel plot. Vertical dotted line shows mean effect size. Black dots individual study effect sizes.

Visual inspection of the funnel plot (Figure 4) and the result of the Egger’s test of the intercept (3.65, 95% CI [– 0.97, 8.26], *p* = .145) provide no evidence of publication bias. Heterogeneity between studies suggests a potential impact of moderator variables on the reported association between diurnal preference and conscientiousness. However, sub-group meta-analyses conducted based on sample composition (students *vs*. general population, Table 2) did not significantly explain heterogeneity in effect size. Similarly, neither age (β = –.006, *p* = .32, 95% CI [– 0.02, 0.01], R^2^ = 0) or sample size (β = 0, *p* = .98, 95% CI [– 0.003, 0.003], R^2^ = 0) or sex ratio of the sample (β = 0, *p* = .78, 95% CI [– 0.007, 0.009], R^2^ = 0) were related to the observed association between diurnal preference and conscientiousness.

**Table 2:** Q-test for between study heterogeneity, p value for subgroup differences.


SUBGROUP ANALYSES	DESCRIPTION	CONTRIBUTING EFFECT SIZES	FISHERS *Z*	95% CI		Q(1)	*P* VALUE

Sample composition	General population	5	0.27	0.19	0.35		

	Students	8	0.31	0.21	0.41	0.49	0.49


### Survey

**Figure 5:**
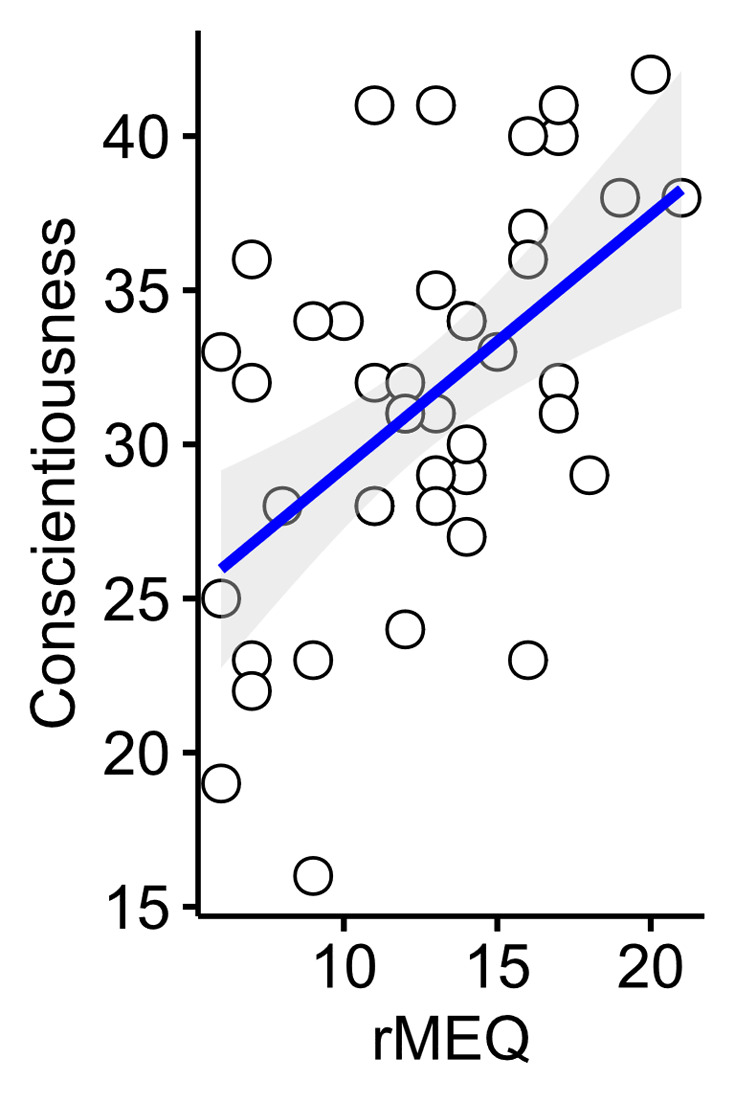
Scatter plot showing the relationship between conscientiousness and diurnal preference. Grey shaded areas shows standard error.

Both rMEQ and Conscientiousness variables were considered approximately normally distributed (*W* = .97, *p* = .27, *W* = .98, *p* = .48) Diurnal preference was positively correlated with the personality trait conscientiousness (*r*(43) = .53, *p* < .001) – please see scatter plot presented as Figure 5.

### Resting fMRI

**Figure 6:**
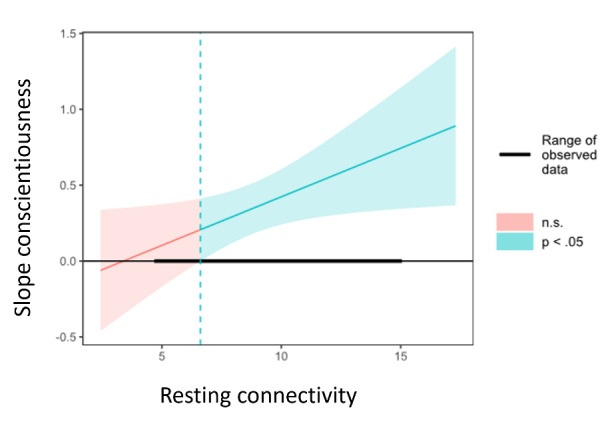
Johnson-Neyman plot showing observed values for the moderator (DMN connectivity) and the threshold for significance of the effect of diurnal preference on conscientiousness (blue shaded area).

The interaction between diurnal preference and DMN connectivity was statistically significant (β = 0.16, *t* = 2.14, *p* = .038, *R^2^* = 0.36) and indicated that with higher levels connectivity the relationship between diurnal preference and conscientiousness was greater. Further exploration showed that the Johnson-Neyman point (i.e., the threshold for significance of the effect of diurnal preference on the conscientiousness, see Figure 6 for details) was located at 6.97. That is, for low values of connectivity up to this value, the association between diurnal preference and conscientiousness was not significant while above this point, diurnal preference was a significant predictor of conscientiousness.

## Discussion

The current meta-analysis demonstrated a small to moderate association between diurnal preference and conscientiousness similar in size (0.29) to those reported by Tsauosis [18] and Lepnivich [16] respectively, 0.29 and 0.32. There was no evidence of a publication bias, nor did we observe evidence for a moderating effect of sample-type (general population *vs*. students) or age and sample sex-ratio (as assessed by meta-regression) on the relationship between diurnal preference and conscientiousness. We also observed a similar relationship in the current online survey (*N* = 43). Finally, using resting-state data acquired in the same individuals that completed the online survey, we observed limited evidence that the relationship between diurnal preference and conscientiousness was moderated by functional connectivity within the Default Mode Network.

The current work and previous meta-analyses [16,18] support the notion that a more morning profile is associated higher levels of conscientiousness. More generally, growing evidence suggests that morningness is related to more adaptive personality traits [45] and emotional stability [48,49]. In older adults, morning types are more likely to be in a job classed as professional or intermediate [50] while in younger adults and adolescents morningness has been reported to be associated with better academic achievement [51]. There is also evidence that evening types are perceived as less healthy, lower self-controlled, less motivated and more immature as compared to morning types [52]. Notably, the directionality of the relationship between diurnal preference and conscientiousness cannot be inferred from the current work. Personality could influence diurnal preference/sleep timing by leading individuals to engage with behaviours which impacts sleep timing – an individual low in conscientiousness may, for example, engage more with activities more likely take place in the late evening/night time (e.g. internet gambling). Alternatively, personality could influence diurnal preference/sleep timing through active decisions made by individuals – a person high in conscientiousness may elect to adopt a sleeping pattern that facilitates punctuality and alertness [53].

Resting-state fMRI provides a measure of functional connectivity both between and within neural networks and is indexed by determining synchronicity of Blood Oxygen-Level Dependent (BOLD) fluctuations while participants are at rest. One such network is the DMN, a widely distributed network and implicated in a number of cognitive functions including memory and abstract thought [54]. Exploring the link between brain network connectivity and conscientiousness, Toschi and colleagues [27] observed that conscientiousness was related to higher functional connectivity in DMN and a fronto-parietal network. More specifically, two facets of conscientiousness in particular (dutifulness and achievement) were related to connectivity within the DMN. These data suggest a neural substrate for conscientiousness and, by extension, a potential explanation of the behaviours typically associated with more conscientious individuals such as being more driven and reliable [27]. Similarly, this network may also be preferentially activated during internal focus (e.g. future planning) a facet associated conscientious individuals [27]. We have previously demonstrated that DMN connectivity is positively associated with diurnal preference [32], Facer-Childs and colleagues [55] reported that functional connectivity with regions of the DMN predict higher attentional performance (as indexed by faster reaction times in the psychomotor-vigilance and Stroop tasks). More recently, Wang et al., [56] reported that chronotype was significantly associated functional connectivity between DMN and the ventral attention network (vAN) and the latter shares considerable overlap with a goal-priority network (GPN) described by Rueter and colleagues and related to conscientiousness [57]. The current finding that DMN connectivity moderated the relationship between diurnal preference and conscientiousness adds to this literature. At lower levels of connectivity in the DMN, diurnal preference was not a significant predictor of conscientiousness. However, at higher levels of connectivity the link between diurnal preference and conscientiousness was statistically significant.

This work is not without limitations and these should be taken into consideration when interpreting the findings. Time of scanning was fixed between 10am and 4pm and participants were free to choose any free slot within this time period. We did not, however, set scan times relative to individual wake up times to ensure that participants were in similar circadian phase. This is of importance as a recent rsFMRI study [58] reported changes in a number of metrics related to resting-state connectivity between scans acquired one hour after habitual wakeup (morning; determined using self-report sleep-wake patterns) and again after 10 hours (evening). In addition, we determined diurnal preference using a single brief self-report metric (the rMEQ). We cannot therefore assess the impact of social jetlag (as estimated by the Munich Chronotype Questionnaire [59]). Future studies that fix scan times relative to individual wake times, and account for additional measures such as social jetlag are warranted. Finally, suitably powered longitudinal studies are required to probe causal directions for the observed relationship between diurnal preference and conscientiousness.

In sum, the current work explored the link between diurnal preference and conscientiousness using three complimentary approaches. Meta-analysis demonstrated a small but significant relationship between diurnal preference and conscientiousness. A similar relationship was observed in the current sample using an online survey. Finally, resting state connectivity analysis demonstrated that the relationship between diurnal preference and conscientiousness was moderated by connectivity within the default mode network.
